# Supplementary material for: Magnetic resonance imaging of tumor response to stroma-modifying pegvorhyaluronidase alpha (PEGPH20) therapy in early-phase clinical trials
Source: Sci Rep. 2024 May 21;14:11570. doi: 10.1038/s41598-024-62470-9 (PMC11109088; doi:10.1038/s41598-024-62470-9)
Supplement: Supplementary file 1 — Supplementary Information. [file 41598_2024_62470_MOESM1_ESM.pdf]

Magnetic Resonance Imaging of Tumor Response to Stroma-Modifying Pegvorhyaluronidase alpha (PEGPH20) Therapy in Early-Phase Clinical Trials

Supplementary Materials

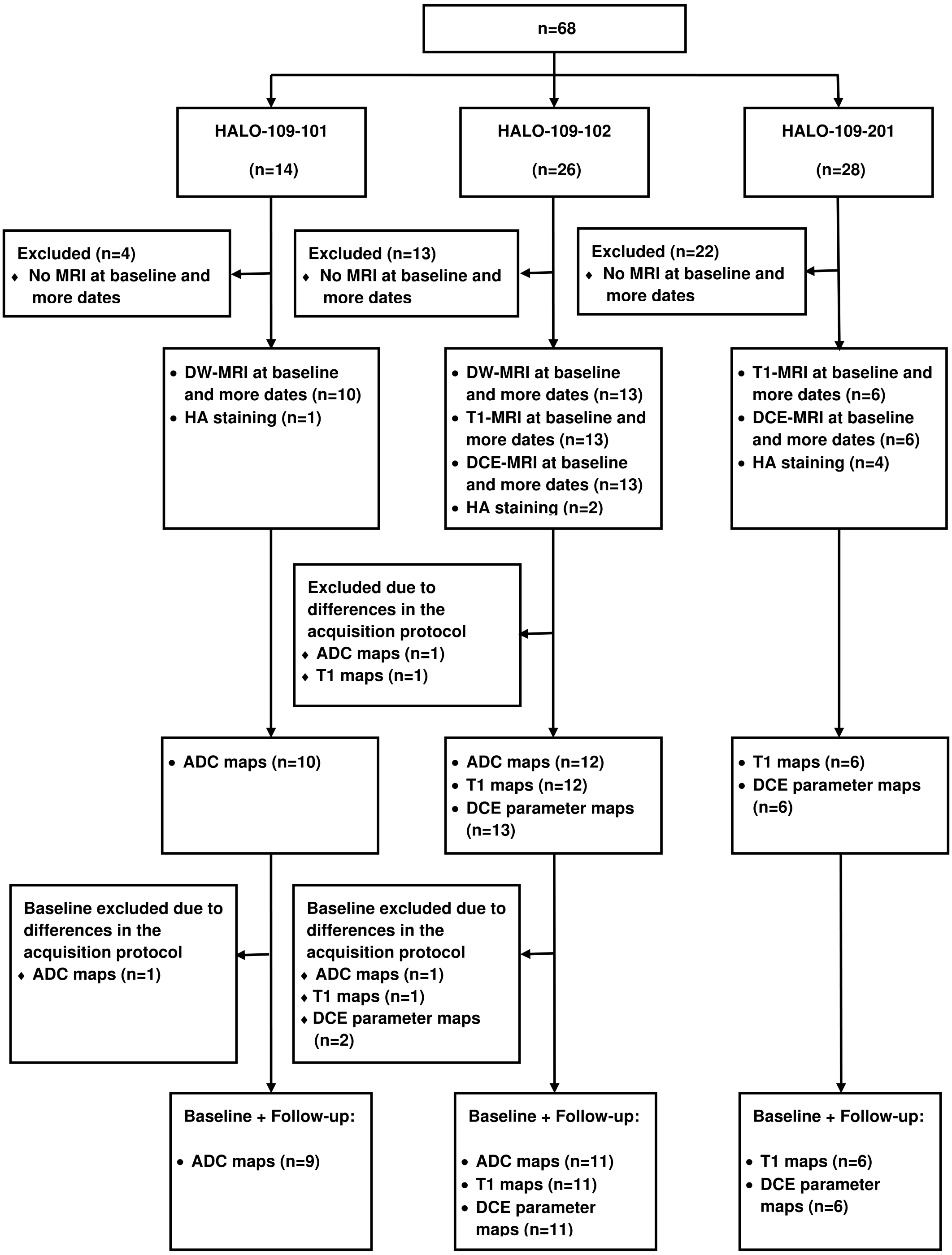

Supplementary Figure 1: CONSORT diagram.

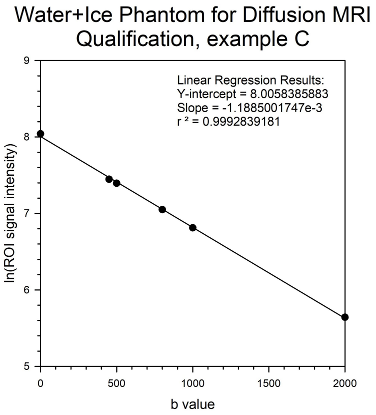

Example A

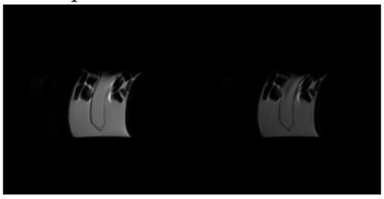

Example B

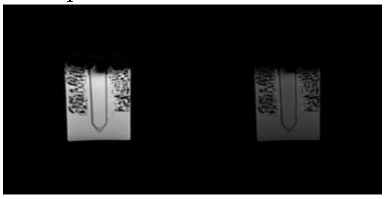

Example C

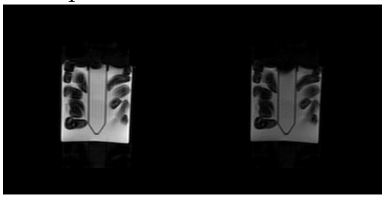

Supplementary Figure 2: Three examples of DW-MRI images of ice-water phantoms acquired at b = 0 and 450 s/mm^2; geometric distortions are visible in Example A (scanner qualification fail). An example of mono-exponential fitting of ADC from ROIs drawn within the inner tube (distilled water) is also shown. Negative slope values outside the range 1.0-1.2 $\times$ 10^{-3} (mm^2/s) constituted a scanner qualification fail [refs 23 and 24], provided the triplicate measurements indicated that phantom temperature was stable during imaging. Follow-up discussions with the site in question were initiated in case of a qualitative (geometric) or quantitative (slope) scanner qualification fail, or if the triplicate measurements suggested that phantom temperature has not stabilized prior to the DW-MRI.

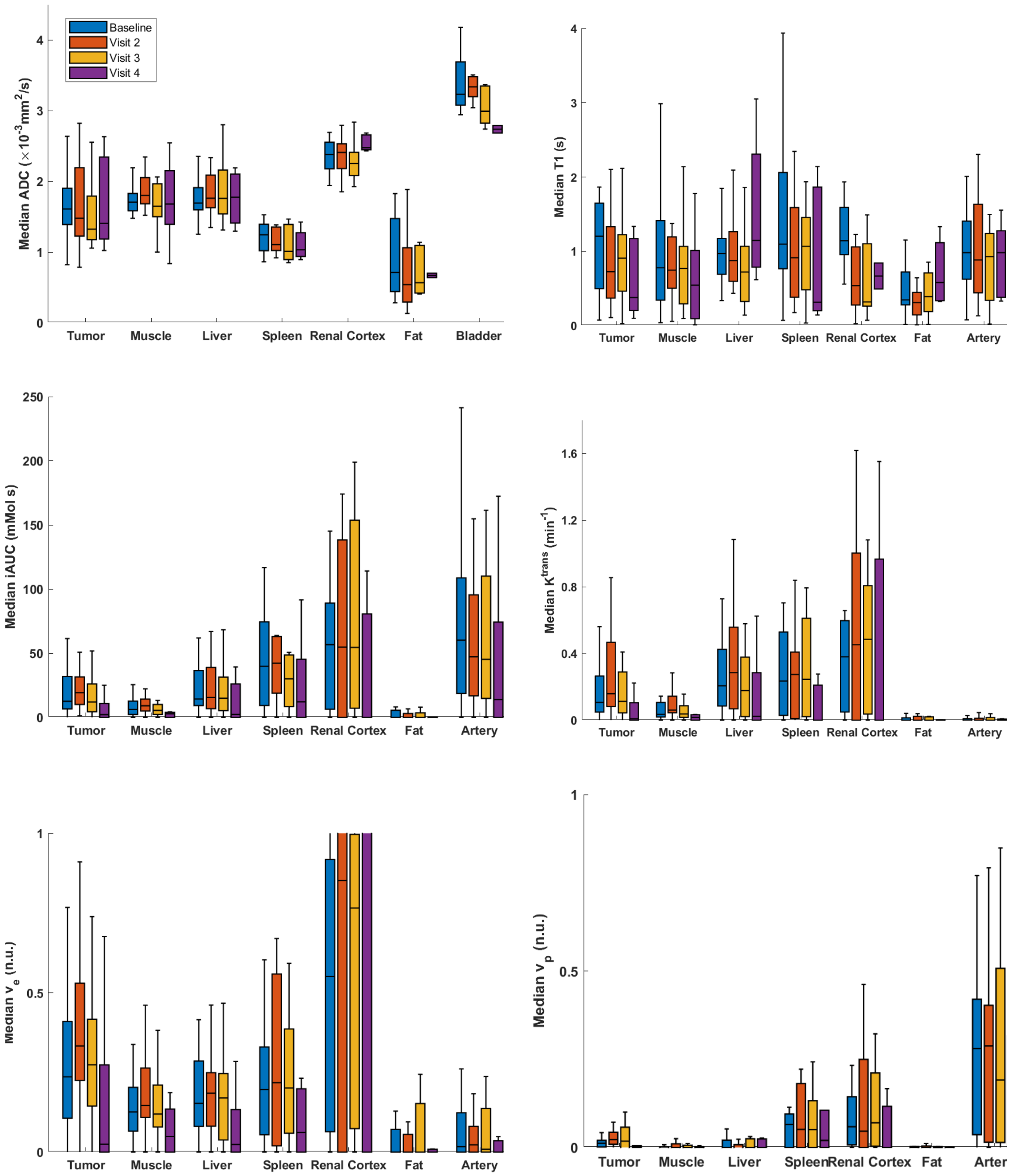

Supplementary Figure 3: Reproducibility analysis for different tissues.

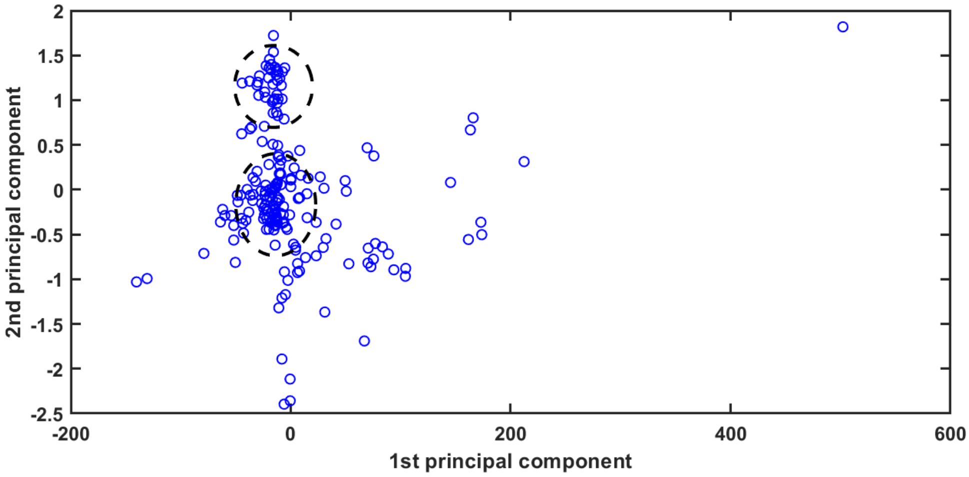

Supplementary Figure 4: Principal component analysis on tumor parameter changes (Day 1 - Baseline) in ADC, T1, iAUC, k^{trans}, v_p, and v_e on 198 voxels equally distributed from 6 patients (102-003-105, 102-003-118, 102-005-111, 102-006-107, 102-006-110, 102-006-124). Projection of the parameter changes in a 2D plane suggests the existence of two clusters manually drawn as dashed circles.

Supplementary Table 1: Summary of patient data pharmacokinetics and response.

	Patient ID	Cmax (U/ml)	Cmin (U/ml)	AUC_{0-72h} (U h/ml)	PFS[1] (Days)	OS[2] (Days)
HALO-109-101	101-001-109	0.7	0.36	1.34	-	-
	101-002-102	31.3	1.83	520	-	-
	101-002-106	0.42	0.42	0.1	-	-
	101-002-114	1.61	0.72	13.5	-	-
	101-003-103	0.48	0.34	5.6	-	-
	101-003-104	-	-	-	-	-
	101-003-105	0.39	0.32	0.99	-	-
	101-003-108	0.53	0.42	1.28	-	-
	101-003-110	0.63	0.52	0.78	-	-
	101-003-112	0.67	0.47	7.38	-	-
HALO-109-102	102-002-102	1.05	0.39	-	-	-
	102-002-103	3.4	0.65	109	-	-
	102-002-113	0.55	0.5	-	-	-
	102-002-115	0.5	0.48	-	-	-
	102-002-126	3.34	0.313	56.5	-	-
	102-003-104	0.74	0.4	6.49	-	-
	102-003-105	0.91	0.4	4.72	-	-
	102-003-117	2.98	0.37	54.6	-	-
	102-003-118	2.81	0.77	96.3	-	-
	102-005-111	1.57	0.625	22.8	-	-
	102-006-107	2.36	0.81	50.2	-	-
	102-006-110	1.56	0.69	14.1	-	-
	102-006-124	1.84	0.53	37.1	-	-
HALO-109-201	201-001-301	1.78	0.53	16.6	159	578
	201-001-304	4.61	0.93	55	52	171
	201-003-306	5.43	1.05	58.6	168	370
	201-007-405	4.63	1.53	45.3	163	176
	201-007-409	3.34	1.03	45.3	225	395
	201-007-414	2.42	0.82	36.8	348	403

[1] PFS: Progression Free Survival
[2] OS: Overall Survival

Supplementary Table 2: Number of patients relative to the total available for each visit with at least one tumor with a significant median parameter change with respect to baseline below and above the repeatability coefficient (RC).

At least one tumor in patient	Visit 2 ADC	Visit 2 T1	Visit 2 iAUC	Visit 2 k^{trans}	Visit 2 v_e	Visit 2 v_p	Visit 3 ADC	Visit 3 T1	Visit 3 iAUC	Visit 3 k^{trans}	Visit 3 v_e	Visit 3 v_p	Visit 4 ADC	Visit 4 T1	Visit 4 iAUC	Visit 4 k^{trans}	Visit 4 v_e	Visit 4 v_p
$< -RC$	4/20	8/17	2/17	0/17	2/17	3/17	4/11	3/15	5/15	1/15	3/15	6/15	2/5	1/6	4/5	3/5	2/5	1/5
$> +RC$	2/20	2/17	9/17	6/17	7/17	7/17	1/11	3/15	3/15	2/15	4/15	5/15	1/5	1/6	1/5	1/5	0/5	1/5

Supplementary Table 3: R^2 (p-value obtained by F-test) between median parameter changes (Day1-Baseline) in tumors and drug dose/pharmacokinetics.

	Dose	Cmax	Cmin	AUC_{0-72h}
ΔADC	0.01 (0.5)	0.01 (0.5)	0 (0.7)	0.01 (0.56)
$\Delta T1$	0.05 (0.27)	0 (0.78)	0.01 (0.61)	0.11 (0.13)
$\Delta iAUC$	0 (0.71)	0 (0.91)	0.02 (0.5)	0.01 (0.59)
Δk^{trans}	0 (0.69)	0 (0.81)	0 (0.99)	0.04 (0.34)
Δv_e	0 (0.96)	0.01 (0.56)	0.07 (0.19)	0.02 (0.51)
Δv_p	0.01 (0.57)	0.03 (0.37)	0 (0.71)	0.02 (0.44)

Supplementary Table 4: R^2 (p-value obtained by F-test) between median parameter changes (Day1-Baseline) in tumors and HA levels in tumor and stroma at baseline. Correlations to changes in HA levels were not obtained as ≤ 2 tumors had the same type of HA measurements before and after PEGPH20.

	HA%	H-score (tumor)	H-score (stroma)
ΔADC	0.05 (0.85)	- (-)	- (-)
$\Delta T1$	- (-)	0.02 (0.84)	0.37 (0.39)
$\Delta iAUC$	- (-)	0.12 (0.65)	0.02 (0.83)
Δk^{trans}	- (-)	0.58 (0.24)	0.64 (0.2)
Δv_e	- (-)	0.09 (0.69)	0.26 (0.49)
Δv_p	- (-)	0.48 (0.31)	0.04 (0.78)

Supplementary Table 5: R^2 (p-value obtained by F-test) between median parameter changes (Day1-Baseline) in tumors and Response by RECIST, PFS and OS.

	RECIST	PFS	OS
ΔADC	- (-)	- (-)	- (-))
$\Delta T1$	0.01 (0.8)	0.12 (0.51)	0.04 (0.67)
$\Delta iAUC$	0.02 (0.76)	0.4 (0.18)	0.1 (0.54)
Δk^{trans}	0.05 (0.65)	0.55 (0.09)	0.26 (0.3)
Δv_e	0.01 (0.79)	0.31 (0.25)	0.21 (0.36)
Δv_p	0.1 (0.53)	0.38 (0.19)	0 (0.99)

Supplementary Table 6: Pixel-wise analysis using a decision tree model. Proportion of p-responses (P%) per tumor, for the training (tr.), and validation (val.) sets are shown (number of pixels per set in parenthesis). Also, it is depicted the proportion of predicted p-responses (PP%) for training, validation and test sets and the BA of the prediction in parenthesis. Average BA per tumor and per patient for the different sets are described in the last two rows.

Patient (tumor)	P%	P% tr.	P% val.	PP% tr.	PP% val.	PP% test
102-006-110	78.6% (467)	50% (26)	80.3% (441)	61.5% (80.8%)	73.4% (70%)	64% (69.7%)
102-003-105 (T1)	100% (11)	100% (7)	100% (4)	85.7% (85.7%)	100% (100%)	100% (100%)
102-003-105 (T2)	86.4% (22)	89.5% (19)	66.7% (3)	94.7% (47.1%)	66.7% (100%)	86.3% (61.4%)
102-003-118	62.2% (9064)	0% (26)	62.4% (9038)	0% (100%)	4% (52.3%)	1.4% (51.1%)
102-005-111	91.8% (171)	84.6% (26)	93.1% (145)	84.6% (85.2%)	77.9% (86.5%)	49.7% (77.1%)
102-006-107	81.2% (239)	19.2% (26)	88.7% (213)	19.2% (50.5%)	46.4% (71.5%)	30.9% (65%)
102-006-124	85.3% (682)	61.5% (26)	86.3% (656)	53.8% (53.1%)	71.1% (66.1%)	73.7% (67.4%)
		Average BA	Per tumor:	71.7%	78%	70.2%
			Per patient:	69.2%	74.4%	65.6%
